# Celiac Disease Patients With Coronary Artery Disease: A Nationwide Population-Based Study

**DOI:** 10.7759/cureus.26151

**Published:** 2022-06-21

**Authors:** Maryam B Haider, Paul Naylor, Avijit Das, Syed M Haider, Murray N Ehrinpreis

**Affiliations:** 1 Internal Medicine, Detroit Medical Center/Wayne State University Sinai-Grace Hospital, Detroit, USA; 2 Gastroenterology, Wayne State University School of Medicine, Detroit, USA; 3 System Science, Binghamton University, Binghamton, USA

**Keywords:** iron deficiency anemia, obesity, type 2 diabetes, type 1 diabetes, hyperlipidemia, hypertension, coronary artery disease, celiac disease

## Abstract

Background

Coronary artery disease (CAD) is associated with celiac disease (CD) with limited evidence. However, the common risk factors linking CD and CAD are still lacking in the literature. Known CAD risk factors include hypertension, hyperlipidemia, type 2 diabetes, obesity, and tobacco use. Common risk factors linking CD and CAD are poorly documented.

Objective

There are three objectives: Firstly, to evaluate potential demographic differences between CD patients with CAD and without CAD. Secondly, to analyze the risk factors of CAD in CD patients. Lastly, to compare CD-CAD and matched non-CD CAD to determine whether there are additional CAD risks in individuals with CD.

Methods

The study is a nationwide retrospective case-control study. The National Inpatient Sample (NIS) database was used to identify patients admitted between 2016 and 2018 with a principal or secondary diagnosis of CD. We analyzed sociodemographic and clinical risk factors of CAD in CD patients and compared the CD-CAD population with the matched non-CD CAD cohort.

Results

Out of 23,441 hospitalizations with CD in 2016-2018, 4244 (18%) were found to have CAD. Established CAD risk factors identified in CD patients included hypertension, hyperlipidemia, type 2 diabetes, and a family history of CAD. In contrast, tobacco use is not a CAD risk factor in CD patients. Female patients with CD had 55% lesser odds of CAD than male patients. The odds of CAD in CD patients with hyperlipidemia were five times higher, 1.2 times higher with essential hypertension, and two times higher with type 2 diabetes. Patients with CAD had a higher prevalence of iron deficiency anemia (9.33% CD-CAD and 8.28% non-CAD CD Vs. 7.32% non-CD CAD).

Conclusions

Our study confirms that, as with non-CD individuals, males and the White race are at increased CAD risk in the CD population. CD-CAD patients have a higher hyperlipidemia prevalence than non-CD CAD patients. CD patients with type 1 diabetes have an early diagnosis of CAD compared to CD patients with type 2 diabetes. Iron deficiency anemia is a statistically significant risk factor for CAD in CD patients.

## Introduction

Celiac disease (CD) is an autoimmune disease caused by a gluten reaction characterized by mucosal inflammation, villous atrophy, crypt hyperplasia of the small bowel, and other extensive systemic manifestations. The prevalence of CD is approximately 0.7% (biopsy confirmed) and 1.4% (serology confirmed) globally [1]. In the 21st century, the pooled incidence of CD among females was 17.4 (95% CI: 13.7, 21.1) per 100,000 person-years, compared with 7.8 (95% CI: 6.3, 9.2) among males [2]. Approximately 1% of the western population suffers from CD [3]. The prevalence of CD is not limited to Europeans. Increasing numbers of CD patients have been seen in Northern Africa, the Middle East, India, and Northern China [4].

The most common gastrointestinal symptoms in response to gluten include chronic diarrhea, malabsorption, unexpected weight loss, abdominal pain, and distension. To establish the diagnosis, patients with the symptoms mentioned above should have positive celiac serology and duodenal biopsy samples showing increased intraepithelial lymphocytes with crypt hyperplasia (Marsh type 2) or, more commonly, also with villous atrophy (Marsh type 3) [5].

Coronary artery disease (CAD) is the leading cause of death from cardiovascular disease in the United States (US). Approximately 18.2 million adults above age 20 are affected, and one death occurs every 36 seconds [6]. Studies by Gajulapalli and Pattanshetty [7] and Emilsson et al. [8] suggest that CD patients are at higher risk of CAD even on a gluten-free diet. However, some studies are not in line with these observations [9,10]. Patients with CD and CAD have other risk factors that might factor into the disease. Gajulapalli and Pattanshetty [7] reported a two-fold increase in the prevalence of CAD in CD patients compared to non-CD patients. They hypothesized that chronic low-level gut inflammation and the autoimmune nature of CD could cause an increase in atherosclerosis. Many studies have shown that cardiovascular diseases enhance the risk of mortality in CD patients [11]. Moreover, Emilsson et al. [8] concluded that ischemic heart diseases are increased in CD patients in the absence of traditional risk factors like elevated cholesterol levels, BMI, and smoking. They proposed that CD's systemic inflammation can itself be a potential risk factor as a 12 % higher C-reactive protein was noticed in CD patients suffering from myocardial infarction.

CAD has been associated with risk factors such as hypertension, hyperlipidemia, diabetes mellitus, obesity, and smoking [12]. On the other hand, in CD patients, hypertension and hyperlipidemia are not significant risk factors [13]. Moreover, iron deficiency anemia may also complicate CD patients [14]. In some CD patients, iron deficiency has been associated with adverse outcomes in CAD [15]. 

Few studies have explored the risk factors for CAD in CD patients. Therefore, the purpose of this paper is three-fold. Firstly, to explore the demographic differences between CD patients with and without CAD. Secondly, to identify CAD risk factors in CD patients. Lastly, to determine the differences in CAD risk factors in CD-CAD compared to non-CD CAD Patients. 

## Materials and methods

Data source and study population

The study is a nationwide retrospective analysis of records obtained from the National Inpatient Sample (NIS) database. The NIS is a member of the databases developed for the Healthcare Cost and Utilization Project (HCUP-NIS) by the Agency for Healthcare Research and Quality [16]. It is the largest publicly available all-payer inpatient database in the US, containing a 20% stratified sample of all discharges from US Hospitals. The NIS database is sampled from the state's inpatient database (SID) from 47 states, plus the District of Columbia, covering 97% of the US population. The publicly accessible HCUP-NIS is a limited de-identified database with discharge records, including demographics, diagnoses, procedures, hospital characteristics, and charges [17]. International Classification of Diseases, Tenth Revision, Clinical Modification (ICD-10-CM) coding provided 30 to 40 diagnoses depending on each patient’s admission year. As the dataset is publicly available, institution review board approval (IRB) was not required from Wayne State University. We selected the NIS data from January 2016 to December 2018, and it was analyzed in the retrospective cohort method.

Model design

All patients diagnosed with CD aged > 17 years were included in this study. We used the ICD-10-CM code of K900 to identify CD patients. To have a larger CD cohort, we included patients admitted with both primary and secondary CD diagnoses. The patient population was split into case and control groups by the presence or absence of CAD. CD-CAD patients were established as cases, and non-CAD CD patients were established as the control group. We selected a non-CD CAD cohort with the 1:1 fixed ratio nearest neighbor (greedy) propensity score method using the patient’s age, gender, and race. CAD included atherosclerotic heart disease, unstable angina, non-ST elevation myocardial infarction, and ST-elevation myocardial infarction.

Patient attributes of interest were age, sex, race, socioeconomic status, and primary health insurance payer. Patient age was categorized into three groups: 18-40, 41-64, and 65 and older, previously defined [18]. The race was categorical (White, Black, Hispanic, Asian, Pacific Islander, Native American, and other), sex was binary (male or female), and all established risk factors were binary (present or not present). Established risk factors including hyperlipidemia, essential hypertension, type 1 diabetes, type 2 diabetes, body mass index (BMI), iron deficiency anemia, family history of CAD, and tobacco are used in this study. We utilized Elixhauser comorbidity software to assess the patients' Elixhauser comorbidity index (ECI). The ECI tool was developed as part of the HCUP, a Federal-State-Industry partnership sponsored by the Agency for Healthcare Research and Quality. ECI is a technique of identifying pre-existing conditions from ICD codes that substantially impact the outcomes, such as hospital length of stay or in-hospital mortality [19].

Statistical analysis

Statistical analyses were performed in RStudio 1.4 (RStudio, Boston, MA). Data is presented in percentages (nominal variables) and mean ± standard deviation (continuous variables). The univariate analysis has been conducted for differences with the student t-test for continuous variables and Pearson’s chi-squared test for nominal variables. All hypothesis tests are two-tailed with a significance level of 0.05. We used regression analysis to estimate the relationship between primary outcome (CAD) and independent variable(s). Univariate linear regression was used for the continuous variables or logistic regression for the dichotomous variables to calculate the primary outcome's unadjusted odds ratio. Furthermore, multivariate logistic regression was conducted to estimate the adjusted odds ratio and analyze the potential risk factors of CAD.

Demographic variables analyzed included patient demographics, age, sex, race, and established risk factors. A multivariate model was constructed from stepwise logistic regression with a significance level of entry of 0.15 and a significance level of stay of 0.10. The model was evaluated for the goodness of fit with the Pearson chi-square and Hosmer-Lemeshow (HL) test. Adult patients (age >17 years) have been included in the analysis. Missing data was labeled as “other” or “unknown.”

## Results

Comparison of patient characteristics and demographics in CD patients, with and without CAD

Table 1 shows the summary of CD patients’ demographics. We identified 23,441 patients admitted to the hospital with CD from NIS data from January 2016 to December 2018. Of these, 4244 (18%) patients identified with CAD in the case group and 19,197 (82%) patients without CAD in the control group. CD was more prevalent in females 72% vs. 28% (male). The age comparison shows that older patients with a mean age of 71.5 (±12.9) years are present in the case group (CD with CAD) as compared to the mean age of 53.4 (±19.9) years in the control (CD) group. Further categorization of the age showed that CAD is most prevalent in patients with age >=65 years (72% were CD with CAD vs. 33% without CAD) as compared to the patients aged 18-40years (2% were CAD with CD vs. 32% without CAD), and patients aged of 41-64 years (26% CAD with CD vs. 34% without CAD).

**Table 1:** Comparison of celiac disease patients, with and without CAD ^1^ Two sample Student t-test, 2-tailed for comparing means of two continuous variables. ^2^ Pearson Chi-Square 2-tailed test for the association of two categorical variables. ^3^ Pearson Chi-square, 2-tailed test for two by n table. Statistical significance illustrates that the two groups differ. CAD: coronary artery disease; ECI: Elixhauser comorbidity index; y: years

Variables	Overall (N=23441)	CAD		P-value
		No (n= 19197) 81.89%	Yes (n= 4244) 18.11%	
Year				0.6394^3^
2016	7847 (33.48%)	6400 (33.34%)	1447 (34.10%)	
2017	7679 (32.76%)	6301(33.82%)	1378 (32.47%)	
2018	7915 (33.77%)	6496 (33.84%)	1419 (33.44%)	
Gender				<0.0001^2^
Female	16793 (71.61%)	14450(75.27%)	2343 (55.21%)	
Male	6648 (28.36%)	4747 (24.73%)	1901 (44.79%)	
Age (y), mean (SD)	56.70 (20.05)	53.44 (19.88)	71.45 (12.92)	<0.05^1^
Age groups (y)				<0.0001^3^
18-40	6185 (26.64%)	6098 (32.13%)	87 (2.05%)	
41-64	7562 (33.57%)	6481 (34.14%)	1081 (25.53%)	
>=65	9469 (40.79%)	6402 (33.73%)	3067 (72.42%)	
Race/Ethnicity				<0.0001^3^
White	20076 (85.64%)	16274(84.77%)	3802 (89.59%)	
Black	728 (3.11%)	616 (3.21%)	112 (2.64%)	
Hispanic	996 (4.25%)	886 (4.62%)	110 (2.59%)	
Asian or Pacific Islander	141 (0.60%)	130 (0.68%)	11 (0.26%)	
Native American	75 (0.32%)	67 (0.35%)	8 (0.19%)	
Other	1425 (6.08%)	1224 (6.38%)	201(4.74%)	
Obesity	2910 (12.41%)	2389 (12.44%)	521 (12.28%)	0.7632^2^
ECI, mean (SD)	1.69 (1.27)	1.58 (1.26)	2.19 (1.22)	<0.05^1^
ECI, by category				
<=0	4549 (19.41%)	4273 (22.26%)	276 (6.50%)	
1-3	16846 (71.87%)	13444(70.03%)	3402 (80.16%)	
>=4	2046 (8.73%)	1480 (7.71%)	566 (13.34%)	
Primary payer status				<0.0001^3^
Medicare	11028 (47.05%)	7792 (40.59%)	3236 (76.25%)	
Medicaid	2621 (11.18%)	2405 (12.53%)	216 (5.09%)	
Private	8682 (37.04%)	7999 (41.67%)	683 (16.09%)	
Self-pay	471 (2.01%)	430 (2.24%)	41 (0.97%)	
No charge	36 (0.15%)	33 (0.17%)	3 (0.07%)	
Other	603 (2.57%)	538 (2.80%)	65 (1.53%)	
Median socioeconomic status by national quartiles				<0.0001^3^
0-25	4164 (17.76%)	3296 (17.17%)	868 (20.45%)	
25-50	5677 (24.22%)	4587 (23.89%)	1090 (25.48%)	
50-75	6474 (27.62%)	5314 (27.68%)	1160 (27.33%)	
75-100	6789 (28.96%)	5722 (29.81%)	1067 (25.14%)	
Other	337 (1.44%)	278 (1.45%)	59 (1.39%)	

CAD is more likely to be prevalent in males (45% CAD with CD vs. 25% without CAD) than females (55% CAD with CD vs. 75% CD without CAD). Regarding racial characteristics, Whites are more likely to have CAD (90% CAD with CD vs. 85% without CAD) as compared to blacks (2.6% CAD with CD vs. 3.2% without CAD), Hispanics (2.6% CAD with CD vs. 4.6% without CAD), Asians (0.3% CAD with CD vs. 0.7% without CAD) and Native Americans (0.2% CAD with CD vs. 0.4% without CAD). Patients on Medicare are more likely to have CAD (76% CAD with CD vs. 41% without CAD) as compared to patients with Medicaid (5% CAD with CD vs. 13% without CAD), patients with private insurance (16% CAD with CD vs. 42% without CAD) and self-pay patients (1.0% CAD with CD vs. 2.2% without CAD).

Analysis of risk factors of coronary artery disease in CD patients

Table 2 presents the risk factors of CAD in CD patients. The results showed that CD patients in the CAD cohort are more likely to have metabolic derangement such as hyperlipidemia (60% CAD vs. 21% non-CAD) and type 2 diabetes mellitus (28% CAD vs. 12% non-CAD), more likely to have essential hypertension, more likely to have iron deficiency anemia due to celiac (9% CAD vs. 8% non-CAD).

**Table 2:** Comparison of risk factors for CAD and non-CAD in CD patients ^1^ Two sample Student t-test, 2-tailed for comparing means of two continuous variables. ^2^ Pearson Chi-Square 2-tailed test for the association of two categorical variables. ^3 ^Pearson Chi-square, 2-tailed test for two by n table. Statistical significance illustrates that the two groups differ. CAD: coronary artery diseases; CD: celiac disease; y: years

Risk Factors									
Variables	Overall (n= 23441)			No-CAD (n= 19197)		Yes-CAD^4^ (n= 4244)	P-value		
Hyperlipidemia		6598 (28.15%)	4049 (21.09%)		2549 (60.06%)			<0.0001^2^	
Essential hypertension		7521 (32.08%)	5708 (29.73%)		1813 (42.72%)			<0.0001^2^	
Iron deficiency anemia		1986 (8.47%)	1590 (8.28%)		396 (9.33%)			<0.05^2^	
Type 1 diabetes		1656 (7.13%)	1393 (7.34%)		263 (6.21%)			<0.05^2^	
Age groups (y) - Type 1 diabetes								<0.05^3^	
18-40		1034 (62.44%)	998 (71.64%)		36 (13.69%)				
41-64		448 (27.05%)	303(21.75%)		145 (55.13%)				
>=65		174 (10.51%)	92 (6.60%)		82 (31.18%)				
Type 2 diabetes		3549 (15.14%)	2346 (12.36%)		1177 (27.79%)			<0.0001^2^	
Age groups (y) - Type 2 diabetes								<0.05^3^	
18-40		204 (5.79%)	196 (8.35%)		8 (0.68%)				
41-64		1380 (39.17%)	1040 (44.33%)		340 (28.89%)				
>=65		1936 (55.04%)	1110 (47.31%)		829 (70.42%)				
Tobbaco		2256 (9.62%)	1864 (9.71%)		392 (9.24%)			0.3441^2^	
Family history (CAD)		1260 (5.38%)	890 (4.64%)		370 (8.72%)			<0.0001^2^	

Interestingly, data revealed that type 1 diabetes mellitus is less common in CD patients with CAD than in non-CAD (6.2% CAD vs. 7.3% non-CAD). Age-wise analysis of patients with type 1 diabetes revealed that patients aged 18-40 have the lowest risk of CAD (14% CAD vs. 72% non-CAD), and patients aged 41-64 have the highest risk of CAD (55% CAD vs. 22% non-CAD). Age-wise analysis of patients with type 2 diabetes showed that patients over 65 years have the highest risk of CAD (70% CAD vs. 47% non-CAD) as compared to patients aged 18-40 (0.7% CAD vs. 8.4% non-CAD), and patients aged 41-64 (29% CAD vs. 44% non-CAD).

Table 3 shows the univariate and multivariate regression analyses. The multivariate regression analysis showed that gender, age, race, hyperlipidemia, essential hypertension, type 2 diabetes, and family history of CAD are statistically significant CAD risk factors in CD patients. 

**Table 3:** Univariate and multivariate analysis of CAD in CD patients in the NIS database from 2016 to 2018. ^1^ Univariate logistic regression is performed in SAS software with PROC Logistic. ^2^ Multivariate logistic regression is performed in SAS software with PROC Logistic with stepwise logistic regression with a 0.15 significance level of entry and 0.10 significance level of stay. NS: not statistically significant; aOR: adjusted odds ratio; CAD: coronary artery diseases; CD: celiac disease; y: years; NIS: National Inpatient Sample; NA: not applicable

Coronary Artery Disease	OR (95%CI)	P value^1^	aOR (95%CI)	P-value^2^
	Univariate logistic regression		Stepwise multivariate logistic regression	
Gender, female vs male	0.405 (0.378 – 0.434)	<0.001	0.450 (0.418 - 0.485)	<0.001
Age groups (y)				
18-40	Reference	NA	Reference	NA
41-64	11.69 (9.37 - 14.58)	<0.001	9.167 (7.275 – 11.551)	<0.001
>=65	33.57 (27.05 - 41.66)	<0.001	25.673 (20.366 - 32.364)	<0.001
Race/ethnicity				
White	Reference	NA	Reference	NA
Black	0.778 (0.634 - 0.955)	<0.05	1.079 (0.858 - 1.359)	0.5148
Hispanic	0.531 (0.435 - 0.650)	<0.001	0.862 (0.689 - 1.078)	0.1932
Asian or Pacific Islander	0.362 (0.196 - 0.671)	<0.05	0.459 (0.235 - 0.895)	<0.05
Native American	0.511 (0.245 - 1.065)	0.0731	0.774 (0.342 - 1.748)	0.5371
Hyperlipidemia	5.626 (5.243 - 6.037)	<0.001	4.75 (4.404 – 5.123)	<0.001
Essential hypertension	1.762 (1.646 - 1.887)	<0.001	1.156 (1.071 – 1.248)	<0.001
Iron deficiency anemia	1.140 (1.015 - 1.279)	<0.05	1.132 (0.998 - 1.283)	0.0540
Type 1 diabetes	0.843 (0.736 - 0.965)	<0.05	NA	NS
Type 2 diabetes	2.740 (2.530 - 2.968)	<0.001	1.855 (1.695 – 2.029)	<0.001
Body mass index (BMI)				
<20	1.068 (0.913 - 1.250)	0.4103	NA	NS
20-24.9	1.239 (0.975 - 1.574)	0.0798	NA	NS
25-29.9	1.253 (0.952 - 1.649)	0.1072	NA	NS
>30 (Obesity)	0.985 (0.890 - 1.089)	0.7641	0.735 (0.656 – 0.823)	<0.001
Tobacco	0.946 (0.844 - 1.061)	0.3442	NA	NS
Family History (CAD)	1.965 (1.732 - 2.229)	<0.001	2.084 (1.791 – 2.424)	<0.001

The odds of CAD in the 41-64 age group are nine times higher (aOR, 9.2; 95% CI, 7.3-11.6; P < .001) and 26 times higher (aOR, 25.7; 95% CI, 20.4-32.4; P < .001) in patients aged over 65 than reference group (18-40 years). Female patients have 55% lesser odds of CAD than male patients (aOR, 0.45; 95% CI, 0.41-0.49; P < .001). In terms of race, we found that Asians have 54% lesser odds of CAD than Whites (aOR, 0.46; 95% CI, 0.24 -0.90; P < 0.05); all other races were statistically not significant.

Hyperlipidemia was the most significant clinical risk factor associated with CAD in CD patients. The odds of CAD with hyperlipidemia were five times higher than those without hyperlipidemia (aOR, 4.8; 95% CI, 4.4-5.1; P < .001). Essential hypertension was also associated with an increase odd of CAD (aOR, 1.2; 95% CI, 1.1-1.3; P < .001). The odds of CAD with type 2 diabetes were two times higher than patients without type 2 diabetes (aOR, 1.9; 95% CI, 1.7-2.0; P < .001). The odds of CAD with obesity are 26% lower than patients those who are not obese (aOR, 0.74; 95% CI, 0.66-0.82; P < .001).

A family history of CAD is associated with an increased likelihood of CAD in CD patients. The odds of CAD with a family history of CAD were two times higher than those without a family history of CAD (aOR, 2.0; 95% CI, 1.8-2.4; P < .001).

Comparison of risk factors of CAD with and without CD

Table 4 compares CAD risk factors in CD-CAD and matched non-CD CAD patients. Type 2 diabetes, obesity, and smoking is relatively more common in non-CD CAD cohort than CD-CAD patients: 28% CD vs. 42% non-CD, 12% CD vs. 19% non-CD, and 9% CD vs. 16% non-CD, respectively. On the other hand, hyperlipidemia, type 1 diabetes, iron deficiency anemia, and family history of CAD are more common in CD-CAD Cohort than CAD non-CD population, 60% non-CD vs. 56% CD, 6.0% non-CD vs. 0.9% CD, 9% non-CD vs. 7% CD, and 9% non-CD vs. 7% CD, respectively.

**Table 4:** Comparison of risk factors of CAD in CD-CAD Vs matched non-CD CAD patients in the NIS database from 2016 to 2018. Data are presented as No. (Percentage) of patients unless indicated otherwise. ^1^ Pearson Chi-Square 2-tailed test for the association of two categorical variables. CAD: coronary artery diseases; CD: celiac disease; NIS: National Inpatient Sample

Variables	CD-CAD (n= 4244)	Non-CD CAD (n= 4244)	P-Value
Hyperlipidemia	2549 (60.06%)	2369 (55.82%)	<0.0001^1^
Essential hypertension	1813 (42.72%)	1880 (44.30%)	0.14241^1^
Iron deficiency anemia	396 (9.33%)	225 (7.32%)	<0.0001^1^
Type 1 diabetes	263 (6.21%)	37 (0.87%)	<0.0001^1^
Type 2 diabetes	1177 (27.79%)	1766 (41.61%)	<0.0001^1^
Tobbaco	392 (9.24%)	660 (15.55%)	<0.0001^1^
Obesity	521 (12.28%)	788 (18.57%)	<0.0001^1^
Family History (CAD)	370 (8.72%)	289 (6.81%)	<0.0001^1^

## Discussion

Studies have shown the prevalence of CAD was significantly higher among patients with CD [7,8,20-24] compared to the non-CD patients. In our literature review, we did not come across studies that examine risk factors of CAD in CD patients. We aimed to further elucidate the association between CAD and CD by conducting this study from a large celiac cohort. We also compared the CD-CAD cohort with the age, sex, and race-matched cohort of non-CD CAD to account for confounding factors that could otherwise affect this study.

Our study confirms that males and older individuals are at increased CAD risk in a CD group. This is consistent with the general population data as the male gender and older age raise the risk of CAD and related cardiovascular complications [25]. In the general population, Caucasians also have an increased lifetime incidence of CAD [26]. Our results confirm that Caucasians have more CAD than African Americans, Asians, or Native Americans.

Previous studies showed that type 1 diabetes was markedly over-represented in CD, especially in men, whereas type 2 diabetes prevalence was similar to the general population [27]. We noted similarly that type 2 diabetes is more associated with CAD in older patients. In our study, CD patients with type 1 diabetes were shown to have a statistically significant decrease in CAD development. Upon further breakdown, however, in type 1 diabetes in the age group of 18-40 and 41-64, there is an increase in the diagnosis of CAD compared to type 2 diabetes in the same age group.

Known risk factors for CAD such as hyperlipidemia, essential hypertension, family history of CAD, type 1 diabetes in patients aged 40 or above, and type 2 diabetes in patients aged 65 or above were associated with increased odds of CAD in the CD population. On the other hand, tobacco, obesity, type 1 diabetes in patients aged 40 or below, and type 2 diabetes in patients aged 65 or less were not associated with CAD in CD patients. In the literature review, data showed an increased risk of ischemic heart diseases in CD [7,22,24,28] despite a lack of traditional risk factors, including hyperlipidemia, BMI, and smoking [7,8,25]. Traditionally, especially with central obesity and elevated blood pressure (BP), hyperglycemia has been the greatest risk factor for cardiovascular disease [6]. We found a higher prevalence of hyperlipidemia but paradoxically a lower prevalence of obesity and tobacco in the CD-CAD cohort as compared to matched non-CD CAD group. Our study results align with Emilsson et al. [8], who found that ischemic heart disease was more common in CD despite having a favorable classic cardiac risk profile (e.g., hypertension, smoking, and obesity). In our study, the odds of CAD with obesity are 26.5% less than patients who are not obese (aOR, 0.735; 95% CI, 0.656-0.823; P <.001). Dietary restructuring as part of CD treatment might contribute to lower BMI. Gajulapalli et al. [7] demonstrated CD patients with increased odds of having hypertension in their study compared to non-CD. We also found that non-CD CAD patients have the highest prevalence of hypertension. Iron deficiency anemia is higher in CD patients as compared to non-CD patients. CD can damage the small intestine, which leads to reduced nutrient absorption and can result in iron deficiency anemia [29]. Schrage et al. demonstrated in three European-based cohorts that absolute and functional iron deficiency anemia is associated with coronary heart disease [30]. We found that patients with CAD have a higher prevalence of iron deficiency anemia (9.33% CD-CAD and 8.28% non-CAD CD Vs. 7.32% non-CD CAD), and it is an independent risk factor of CAD in CD patients.

There are multiple limitations in using the NIS dataset, including the inability to access laboratory values, treatment options given to patients, testing conditions, compliance with gluten-free diets, and exact biopsy results. This study is performed on the inpatient population. As CD is generally an outpatient diagnosis, many patients being outpatients were excluded from our study. While using the administrative database, we were dependent on using ICD 10 codes for all the diagnoses. It is well known that the accuracy of ICD codes carries extreme importance for the study to be reliable. Coding errors might be unavoidable in this situation. As specific identifiers are not being used while using any database, there may be a chance of counting the same patient multiple times in our analysis. Inherent database limitations also include a lack of disease process-specific variables and coding errors without formal validation.

Our study's strength is that our patient population includes almost all patients admitted with CD diagnosis in the US, giving us a large cohort of 23,441 CD patients. To the best of our knowledge, this is the first nationwide study from the NIS database that analyzes sociodemographic and clinical risk factors of CAD in CD and non-CD patients and compares the CD-CAD cohort, non-CD CAD, and non-CAD CD patients. 

## Conclusions

Our study confirms that, as with non-CD individuals, males and the White race are at increased CAD risk in the CD population. Established CAD risk factors identified in CD patients included hypertension, hyperlipidemia, type 2 diabetes, and a family history of CAD. In contrast, obesity and tobacco use are not CAD risk factors in CD patients. CD-CAD patients have a higher prevalence of hyperlipidemia compared to non-CD CAD patients. Furthermore, we noted that CD patients with type 1 diabetes have an early diagnosis of CAD compared to CD patients with type 2 diabetes. Finally, we found iron deficiency anemia as a statistically significant risk factor for CAD in CD patients.

## References

[1] Singh P, Arora A, Strand TA (2018). Global prevalence of celiac disease: systematic review and meta-analysis. Clin Gastroenterol Hepatol.

[2] King JA, Jeong J, Underwood FE (2020). Incidence of celiac disease is increasing over time: a systematic review and meta-analysis. Am J Gastroenterol.

[3] Ludvigsson JF, Card TR, Kaukinen K, Bai J, Zingone F, Sanders DS, Murray JA (2015). Screening for celiac disease in the general population and in high-risk groups. United European Gastroenterol J.

[4] Ashtari S, Najafimehr H, Pourhoseingholi MA (2021). Prevalence of celiac disease in low and high risk population in Asia-Pacific region: a systematic review and meta-analysis. Sci Rep.

[5] Fry L, Seah PP, McMinn RM, Hoffbrand AV (1972). Lymphocytic infiltration of epithelium in diagnosis of gluten-sensitive enteropathy. Br Med J.

[6] Virani SS, Alonso A, Benjamin EJ (2020). Heart disease and stroke statistics-2020 update: a report from the American Heart Association. Circulation.

[7] Gajulapalli RD, Pattanshetty DJ (2017). Risk of coronary artery disease in celiac disease population. Saudi J Gastroenterol.

[8] Emilsson L, Carlsson R, Holmqvist M, James S, Ludvigsson JF (2013). The characterisation and risk factors of ischaemic heart disease in patients with coeliac disease. Aliment Pharmacol Ther.

[9] West J, Logan RF, Card TR, Smith C, Hubbard R (2004). Risk of vascular disease in adults with diagnosed coeliac disease: a population-based study. Aliment Pharmacol Ther.

[10] Jansen H, Willenborg C, Schlesinger S (2015). Genetic variants associated with celiac disease and the risk for coronary artery disease. Mol Genet Genomics.

[11] Viljamaa M, Kaukinen K, Pukkala E, Hervonen K, Reunala T, Collin P (2006). Malignancies and mortality in patients with coeliac disease and dermatitis herpetiformis: 30-year population-based study. Dig Liver Dis.

[12] Malakar AK, Choudhury D, Halder B, Paul P, Uddin A, Chakraborty S (2019). A review on coronary artery disease, its risk factors, and therapeutics. J Cell Physiol.

[13] Sari C, Bayram NA, Doğan FE (2012). The evaluation of endothelial functions in patients with celiac disease. Echocardiography.

[14] Schuppan D, Junker Y, Barisani D (2009). Celiac disease: from pathogenesis to novel therapies. Gastroenterology.

[15] Salardi S, Maltoni G, Zucchini S (2017). Whole lipid profile and not only HDL cholesterol is impaired in children with coexisting type 1 diabetes and untreated celiac disease. Acta Diabetol.

[16] (2021). HCUP: Overview of the National (Nationwide) Inpatient Sample (NIS). http://www.hcup-us.ahrq.gov/nisoverview.jsp.

[17] (2021). HCUP: Clinical Classifications Software (CCS) for ICD-9-CM. http://www.hcup-us.ahrq.gov/toolssoftware/ccs/ccs.jsp.

[18] Ciprandi G, Ruffoni S, Tosca M, Minetti I, Dellepiane S (2011). Asthma and COPD exacerbations: an 8 year survey. Eur J Intern Med.

[19] Elixhauser A, Steiner C, Harris DR, Coffey RM (1998). Comorbidity measures for use with administrative data. Med Care.

[20] Heikkilä K, Koskinen OA, Agarwal A, Tikkinen KA, Mäki M, Kaukinen K (2015). Associations of coeliac disease with coronary heart disease and cerebrovascular disease: a systematic review and meta-analysis. Nutr Metab Cardiovasc Dis.

[21] Emilsson L, James S, Ludvigsson JF (2014). Ischaemic heart disease in first-degree relatives to coeliac patients. Eur J Clin Invest.

[22] Ludvigsson JF, de Faire U, Ekbom A, Montgomery SM (2007). Vascular disease in a population-based cohort of individuals hospitalised with coeliac disease. Heart.

[23] Ciaccio EJ, Lewis SK, Biviano AB, Iyer V, Garan H, Green PH (2017). Cardiovascular involvement in celiac disease. World J Cardiol.

[24] Ludvigsson JF, James S, Askling J, Stenestrand U, Ingelsson E (2011). Nationwide cohort study of risk of ischemic heart disease in patients with celiac disease. Circulation.

[25] Andrikopoulos GK, Mela EK, Georgakopoulos CD, Papadopoulos GE, Damelou AN, Alexopoulos DK, Gartaganis SP (2009). Pseudoexfoliation syndrome prevalence in Greek patients with cataract and its association to glaucoma and coronary artery disease. Eye (Lond).

[26] Meadows TA, Bhatt DL, Cannon CP (2011). Ethnic differences in cardiovascular risks and mortality in atherothrombotic disease: insights from the Reduction of Atherothrombosis for Continued Health (REACH) registry. Mayo Clin Proc.

[27] Kylökäs A, Kaukinen K, Huhtala H, Collin P, Mäki M, Kurppa K (2016). Type 1 and type 2 diabetes in celiac disease: prevalence and effect on clinical and histological presentation. BMC Gastroenterol.

[28] Ozturk K, Altun B, Kurt O (2015). Arterial stiffness in patients with celiac disease. Eur J Gastroenterol Hepatol.

[29] Rubio-Tapia A, Barton SH, Rosenblatt JE, Murray JA (2009). Prevalence of small intestine bacterial overgrowth diagnosed by quantitative culture of intestinal aspirate in celiac disease. J Clin Gastroenterol.

[30] Schrage B, Rübsamen N, Ojeda FM (2021). Association of iron deficiency with incident cardiovascular diseases and mortality in the general population. ESC Heart Fail.

